# Inclusion of Community-Based Participatory Research in High-Impact Medical Journals

**DOI:** 10.1089/heq.2024.0151

**Published:** 2025-05-27

**Authors:** Michelle Doughtery, Yesmina Salib, Mylynda Massart, Jaime E. Sidani, Jean L. Raphael, Felicia Savage Friedman, Jason Beery, Monica Ruiz, Elizabeth Miller, Maya I. Ragavan

**Affiliations:** ^1^ Department of Behavioral and Community Health Sciences, School of Public Health, University of Pittsburgh, Pittsburgh, Pennsylvania, USA.; ^2^ Division of General Academic Pediatrics; University of Pittsburgh and UPMC Children’s Hospital of Pittsburgh, Pittsburgh, Pennsylvania, USA.; ^3^ Division of General Academic Pediatrics Department of Family Medicine, School of Medicine, University of Pittsburgh, Pittsburgh, Pennsylvania, USA.; ^4^ Department of Pediatrics, Baylor College of Medicine, Houston, Texas, USA.; ^5^ YogaRoots On Location, Pittsburgh, Pennsylvania, USA.; ^6^ Equitable and Just Greater Pittsburgh, Pittsburgh, Pennsylvania, USA.; ^7^ Casa San Jose, Pittsburgh, Pennsylvania, USA.; ^8^ Division of Adolescent and Young Adult Medicine, University of Pittsburgh and UPMC Children’s Hospital of Pittsburgh, Pittsburgh, Pennsylvania, USA.; ^9^ Clinical and Translational Science Institute, University of Pittsburgh, Pittsburgh, Pennsylvania, USA.

**Keywords:** community-based participatory research, participatory action research, community partnership

## Abstract

Community-based participatory research (CBPR) is essential for addressing health care inequities; however, it is unclear to what extent articles published in high-impact medical journals use CPBR. We reviewed original research articles in nine journals across 4 years to determine how frequently CBPR was used and, for articles using CBPR, details about partnerships. Of 5,624 articles, only 6 (0.1%) used CBPR. Five identified community partners and whether partners were involved in research planning/implementation, one reported that partners were involved in dissemination, and none reported adherence to CBPR principles. Improving integration of CBPR is an urgent priority for funders, institutions, journals, and researchers.

## Introduction

On the spectrum of community-engaged research approaches, community-based participatory research (CBPR) most closely involves community members as collaborators through equitable partnership, power sharing, and reciprocity.^1^ Key principles of CBPR include recognizing community as a unit of identity, building on community strengths and resources, partnering collaboratively through all phases of the research process, integrating knowledge and action to the mutual benefit of all partners, countering power imbalances, and promoting co-learning through a cyclical, iterative process.^2^ Multiple systematic reviews have documented how CBPR increases recruitment and retention among populations with marginalized identities, facilitates attainment of study goals, and fosters community capacity and action to advance health equity.^3–5^

Given persistent inequities in health and health care affecting marginalized communities, there is an urgent need to incorporate CBPR approaches into medical research.^6^ Leaders of health care organizations, payers, and accreditors across the U.S. have prioritized dismantling health disparities and promoting equity, further underscoring the need for CBPR in medical research.^7^ However, there are numerous institutional barriers to conducting CBPR in academic health care settings, underscoring the importance of evaluating the extent to which these approaches are being used by researchers in the medical field.^8^ Our objectives were to: (1) assess inclusion of articles explicitly using CBPR in high-impact medical journals; and (2) for articles using CBPR, describe how researchers partnered with community collaborators and the extent to which studies adhered to CBPR principles.^2^

## Methods

To assess how frequently and to what extent CBPR has been used by articles published in the medical field, we used a methodology adapted from Chen et al., where the authors similarly reviewed articles in specific journals to understand how frequently participants who use languages other than English were included.^9^ We first identified a sample of articles that we then screened for CBPR approaches. To create this sample, we selected nine journals based on their emphasis on primary care, high impact in the medical field, and capacity to disseminate best practices: *Annals of Family Medicine*, *Annals of Internal Medicine*, *Journal of the American Board of Family Medicine*, *JAMA*, *JAMA Internal Medicine*, *JAMA Pediatrics*, *The Lancet*, *New England Journal of Medicine*, and *Pediatrics*. We included original research articles published in 2021, 2016, 2011, and 2006 as we anticipated that selecting four equally spaced time points over the past two decades would yield a representative sample of articles.

Articles were screened for the term “community-based participatory research,” “participatory action research,” and additional synonymous terms from other reviews (Table 1).^3–5,10^ For each article, one team member used the find function to search for the terms in the full text of the article (i.e., excluding references). If any of the terms were located, the surrounding text was reviewed to determine if CBPR approaches were used; articles where CBPR was described in some other way (e.g., in the discussion section as a next step) were eliminated. We did not review the reference lists of included articles to identify additional articles, as our goal was to assess the use of CBPR among articles in our sample. A second team member re-reviewed the full text of each article containing search terms to verify the determination made by the first reviewer, adjudicating any discrepancies with the senior investigator.

**Table 1.** Inclusion Search Terms

Key words used in text search
Community based participatory process Community based participatory research Community engaged research Community engaged scholarship Community partnered research Community partnered participatory research Community partnered scholarship Participatory action research Stakeholder engaged research Youth participatory action research

For articles using CBPR, we recorded whether articles identified: (1) their community partners; (2) parts of the research process in which they were involved, consistent with CBPR guidelines (shaping purpose and scope; research implementation; and dissemination);^11^ and (3) how researchers attended to key principles of CBPR.^2^ We assessed the level of detail provided by articles around how partners were incorporated into each stage of the research process (i.e., any details corresponding to items in the aforementioned guidelines) and whether community collaborators were included as co-authors (i.e., by reviewing author affiliations to see if any were associated with nonacademic organizations and/or community partner organization[s]).

## Results

**FIG. 1.**
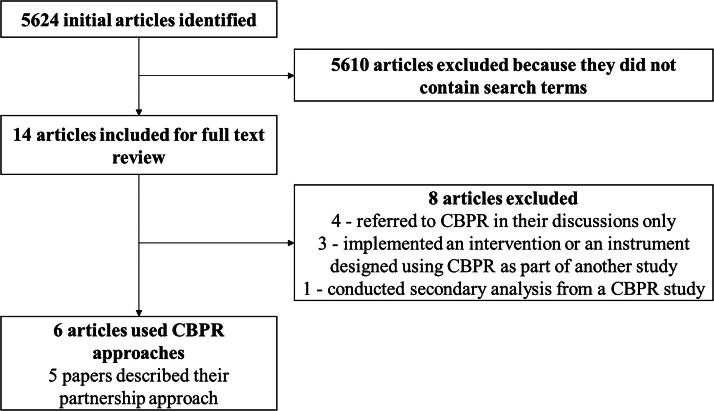
Search Results. Number of articles identified in literature review, number excluded along with reason for exclusion, and final number using community-based participatory research (CBPR) approaches.

There were 5,624 original articles published in identified journals in selected years, of which 14 included at least one of the key CBPR terms (Table 1; Fig. 1). Of these, six (0.1%) reported using CBPR as part of their methods.^12–17^ Four focused on pediatric health, one on women’s health, and one on mental health. Three studies were published in 2006, three in 2016, and none in 2011 and 2021. With this small sample, we were unable to examine trends over time.

Of the six studies using CBPR, 5 identified community partners and specified the stages of research in which they were involved (Table 2).^12–16^ All five indicated whether community partners were involved in planning/design and research implementation processes; only one reported whether partners were involved with dissemination processes. Three studies shared details around how partners were involved in each of these research processes; the other two stated partners were involved without additional detail. Two articles included community collaborators as co-authors, and none explicitly stated how they adhered to principles of CBPR.^2^

**Table 2.** Articles Describing the Role of Community Partners in Their Approach

First author (year), citation number	Journal	Title	Study type	Did the article identify community partners and what stage of the research process they were involved with?				Did the article provide any details on how community partners were involved in these stages of the research process?	
				Identification of partners	Research priorities/ design	Research implementation	Research dissemination		Did the article include community collaborators as co-authors?
Meckler (2006)^13^	*JAMA Pediatrics*	Non-Disclosure of Sexual Orientation to a Physician Among a Sample of Gay, Lesbian, and Bisexual Youth	Cross-sectional	X	X	X			
Reis (2006)^14^	*Pediatrics*	Screening children to identify families at increased risk for cardiovascular disease	Cross-sectional	X	X	X			X
Rosato (2006)^12^	*Lancet*	Women’s groups’ perceptions of maternal health issues in rural Malawi	Qualitative	X	X	X	X	X	X
Cradock (2016)^15^	*JAMA Pediatrics*	Promoting Physical Activity With the Out of School Nutrition and Physical Activity (OSNAP) Initiative	Cluster randomized control trial	X	X	X		X	
Lam (2016)^16^	Journal of the American Board of Family Medicine	The Impact of Community Engagement on Health, Social, and Utilization Outcomes in Depressed, Impoverished Populations: Secondary Findings from a Randomized Trial	Comparative effectiveness research	X	X	X		X	

## Discussion

CBPR is a well-established approach that can help foster research trustworthiness, increase diversity and inclusivity in recruitment and retention, and ultimately disrupt health disparities and promote health equity. We conducted one of the first reviews examining the use of CBPR approaches in high-impact medical journals and found that a minute fraction (0.1%) of articles over 4 years explicitly stated the use of these approaches. This finding comports with a similar review, which found that <1% of peer-reviewed publications in mainstream psychology journals 2004–2014 utilized CBPR.^10^ This percentage is likely higher in other fields where CBPR is more common, such as public health, highlighting the need for a more complete review of CBPR across fields.

Among the few articles identified in our search as using CBPR, only one reported whether community partners were involved in dissemination or use of research findings. Dissemination of research findings to those who could directly benefit is necessary for translating advancements in biomedical science and clinical research into improved population health outcomes.^18,19^ Involving community partners in dissemination can help researchers more fully understand the value of their findings and how to effectively communicate them (e.g., using plain language and visual communication methods).^20,21^ Moreover, community-involved dissemination can counter extractive, one-sided research practices and instead increase capacity sharing between researchers and community members, laying the groundwork for future partnerships.^8,21,22^ Involving community collaborators in manuscripts allows for mutual learning and shared ownership; however, we found that only two articles in our review included community collaborators as co-authors.^12,14^

For articles that used CBPR, the level of detail provided on how partners were involved in research processes was scant and inconsistent. For example, out of the six studies, five identified their partners, and only three described how partners were included in the research process, including using their knowledge and expertise to identify research priorities^12^ or interpreting and informing analysis (e.g., identifying subgroups of interest).^15,16^ Understanding how community partners are involved in research processes can help to assess whether research questions and outputs are valid and meaningful to communities experiencing marginalization, rather than perpetuating biases and unintended assumptions.^23^

Although several articles cited foundational CBPR principles, none specifically described how they attended to these principles. For example, none provided enough detail to assess whether intended study users (i.e., beneficiaries, stakeholders) were adequately represented, whether barriers to participation were addressed, or how partnerships were built and maintained. Crucially, none addressed how trust was established between researchers and community collaborators. Authentic application of CBPR often requires fundamental changes to institutional structures to counter traditional power imbalances between researchers and community members.^8^ Without elucidating how such power imbalances are addressed, it is difficult to assess whether CBPR is applied in name only.^22^

It is possible that study teams in selected articles did indeed adhere to CBPR principles and involve community collaborators in dissemination but did not include these details in their articles. As other reviews have suggested,^4,5^ there is a need for clear reporting standards to guide researchers on how to document community partners’ involvement in research processes and the extent to which studies adhere to CBPR principles.^2^ CBPR guidelines and efforts to measure and evaluate the implementation of CBPR could be a useful starting point for developing such standards.^11,24,25^

We recognize limitations to this study. This review is limited to 4 years of articles in nine journals, missing potential CBPR articles in other years or journals. We intentionally focused on research in high-impact medical journals to understand community-based participatory research within the medical field, which historically has been slower to engage in this work. However, we recognize that we are missing perspectives from journals focused on public health and other clinical fields. Replicating this work with other journals is necessary to elucidate a complete picture of community-based research described in medical, clinical, and public health journals. Additionally, we may have missed studies that incorporated CBPR without using this terminology; however, our goal was to assess articles where authors explicitly described the use of CBPR.

These limitations notwithstanding, results from this review inform multiple implications to advance CBPR in medical research. First, it is important to elucidate why there has been a paucity of published studies using CBPR approaches in selected journals. Researchers using CBPR may favor other journals (e.g., they anticipate a better fit), while high-impact medical journals may tend to accept articles using methods other than CBPR (e.g., because they deem CBPR articles lower quality/rigor). Journal editors should collaborate with CBPR researchers to identify barriers to publishing and strategies for addressing these barriers (e.g., increasing space available for articles using CBPR to thoroughly describe their approach, addressing gaps in reviewers’ understanding of CBPR). Journal editors could develop and publicize guidelines for authors around reporting CBPR research. Additionally, prioritizing CBPR in journal aims and through special calls for CBPR articles could stress the importance of this research approach. Further, there must be institutional investment in CBPR, including training in CBPR; inclusion of community engagement as part of promotion and tenure packages; recognition for nonacademic dissemination (e.g., infographics, data presentations to community partners, data literacy sessions for community organizations); funding to support community partnership development and maintenance; and co-creation and translation of research findings.^8,18,21^

Increasing the prevalence and quality of reporting on CBPR in top medical journals is crucial for closing gaps in the translation of scientific findings, especially to communities burdened by health inequities. This study provides a call to action for researchers, academic institutions, journals, and funders to improve implementation and dissemination of CBPR to a broad readership poised to generate high-impact, actionable science.

## Abbreviations Used

CBPR: Community-based participatory research
